# INK4 cyclin-dependent kinase inhibitors as potential prognostic biomarkers and therapeutic targets in hepatocellular carcinoma

**DOI:** 10.1042/BSR20221082

**Published:** 2022-07-14

**Authors:** Hui Liu, Songhao Jia, Kun Guo, Rongkuan Li

**Affiliations:** 1 Department of Gastroenterology, Second Hospital of Dalian Medical University, Liaoning 116000, China; 2 Department of Pathology, Second Hospital of Dalian Medical University, Liaoning 116000, China; 3 Department of Infectious Diseases, Second Hospital of Dalian Medical University, Liaoning 116000, China

**Keywords:** biomarkers, Hepatocellular carcinoma, immune, INK4, prognosis

## Abstract

The INK4 family is an important family of cyclin-dependent kinase inhibitors (CDKIs) and consists of CDKN2A, CDKN2B, CDKN2, and CDKN2D. Abnormal expression of CDKN2A has been reported in hepatocellular carcinoma (HCC) and is associated with the prognosis of patients and infiltration of immune cells. However, there is a lack of systematic research on the roles of the other INK4 family members in the diagnosis, prognosis, and immune regulation of HCC. Using online public databases and clinical samples, we comprehensively analyzed the INK4 family in HCC. All four INK4 proteins were overexpressed in HCC and correlated with advanced cancer stage and poor prognosis. INK4 expression accurately distinguished tumor from normal tissue, particularly CDKN2A and CDKN2C. The INK4 family participated in cell-cycle regulation and the DNA damage repair pathway, which inhibited genotoxic-induced apoptosis in tumorigenesis. INK4 proteins were positively correlated with the infiltration of immune cells (B cells, CD8+ T cells, CD4+ T cells, macrophages, neutrophils, and dendritic cells) and immune checkpoints (CTLA-4, PD1, and PD-L1). CDKN2D had the highest correlation (correlation coefficient >0.3) with all the above-mentioned infiltrating immune cells and immune checkpoints, indicating that it may be useful as an immunotherapy target. The INK4 family was valuable for diagnosis and predicting the prognosis of HCC and participated in the occurrence, progression, and immune regulation of HCC, demonstrating its potential as a diagnostic and prognostic biomarker and therapeutic target in HCC.

## Introduction

Hepatocellular carcinoma (HCC) is one of the most common malignant tumors in many areas worldwide, with high incidence and mortality [1]. HCC is the fifth most common cancer in men and seventh most common cancer in women and is responsible for over half a million deaths annually [1–5]. Because of the limited therapeutic options and high rate of recurrence and metastasis, HCC is among the most intractable and lethal diseases [2,6]. Additionally, in many patients, HCC is not detected until advanced stages, when the treatment effect and prognosis of patients are very poor [7]. Although some biomarkers of HCC have been reported, related studies are mostly in the preliminary stage. Thus, exploring alterations in genes and molecules may improve the screening, diagnosis, and therapeutic strategies for liver cancer.

Cyclin-dependent kinase inhibitors (CDKIs), which negatively regulate the cell cycle by binding to the cyclin-CDK complex, belong to the INK4 family [8,9]. In addition to functioning as CDK inhibitors, the INK4 family has also been implicated to play important roles in promoting resistance to apoptosis and improving DNA repair, leading to enhanced genome stability and cellular survival under conditions of genotoxic stress [8,10]. The INK4 family consists of CDKN2A (p16^INK4a^), CDKN2B (p15^INK4b^), CDKN2C (p18^INK4c^), and CDKN2D (p19^INK4d^) [8,11]. Abnormal expression of CDKN2A has been reported in HCC and is associated with prognosis and infiltrating levels of immune cells [12]. However, the role of the other INK4 family members, namely CDKN2B, CDKN2C, and CDKN2D, in the diagnosis, prognosis, and immune regulation of HCC is unclear. Therefore, in the present study, we comprehensively analyzed the expression of INK4 family members and their diagnostic and prognostic values, as well as their correlation with the tumor immune microenvironment and immune checkpoints in HCC.

## Methods

### Analysis of INK4 expression using online public databases

We evaluated the expression of the INK4 family in 20 cancer types based on ONCOMINE (www.ONCOMINE.org) [13], which is a publicly accessible online cancer microarray database. Differences in gene expression were compared using Student’s *t*-test. Cutoff values for the *P*-value and fold-change were as follows: *P*-value<0.01, fold-change > 1.5, data type: mRNA. Based on The Cancer Genome Atlas (TCGA) database (https://portal.gdc.cancer.gov/) [14], we further compared the expression of the INK4 family in HCC and adjacent tissues using the R ggplot2 package and evaluated its diagnostic value through receiver-operating characteristic (ROC) curves using the pROC package.

### Clinical verification of INK4 expression

Quantitative real-time polymerase chain reaction (qRT-PCR) and immunohistochemical staining were performed to confirm the expression of INK4 in HCC and adjacent tissues. Thirty patients diagnosed with HCC were randomly selected from the Second Hospital of Dalian Medical University, and patient information is presented in Supplementary Table S1. RNA was extracted from both HCC and adjacent tissues for cDNA amplification and qRT-PCR using the ThermoScript RT-PCR system (Invitrogen, Carlsbad, CA, USA) and StepOnePlus apparatus (Applied Biosystems, Foster City, CA, USA). Glyceraldehyde-3-phosphate dehydrogenase (GAPDH) was used as an internal reference gene. The primer sequences for GAPDH and INK4 family members are listed in Supplementary Table S2. Immunohistochemical staining was performed to validate INK4 expression in HCC tissues. The following antibodies were used as primary antibodies: CDKN2A (ab108349, Abcam, Cambridge, UK), CDKN2B (AF0230, Affinity Biosciences, Cincinnati, OH, USA), CDKN2C (ab192239, Abcam), and CDKN2D (10272-2-AP, Proteintech, Rosemont, IL, USA). Goat antimouse Alexa fluor SP-9000 from ZSGB Biotechnology (Beijing, China) was used as a secondary antibody.

### Correlation analysis between INK4 and cancer stage and prognosis

Correlation analysis between INK4 and cancer stage, including T stage and pathological stage, was performed with R using the ggplot 2 package based on TCGA dataset. The Kaplan–Meier plotter (www.kmplot.com) [15] was used to evaluate the prognostic value of mRNA expression in the INK4 family. Patients with HCC were determined by dividing patient samples into two groups based on the best performance threshold; the prognostic indicators included overall survival (OS), progression-free survival (PFS), and disease specific survival (DSS).

### Gene Ontology and Kyoto Encyclopedia of Genes and Genomes enrichment analyses

Before functional enrichment analyses, the coexpressed genes of INK4 with Pearson correlation coefficients (r) of |r| > 0.4 and *P*<0.001 were obtained with R using start packages based on TCGA database. Gene Ontology (GO) and Kyoto Encyclopedia of Genes and Genomes (KEGG) analyses were performed on the top 200 coexpressed genes with the R package ‘clusterProfiler’ to explore the possible biological functions and signaling pathways affected by the INK4 family. GO analysis included biological process, cell composition, and molecular function. The intersection between coexpressed genes was selected for further functional enrichment analysis to explore their common biological functions and signaling pathways using a Venn diagram. A protein–protein interaction network highlighting the intersection of coexpressed genes of INK4 was constructed using the STRING (string-db.org) database [16]. The obtained protein–protein interaction network was imported into the Cytoscape software, and ten hub genes were selected using CytoHubba [17]. Kaplan–Meier plots were generated, and the log-rank test was performed using the survival package to explore the relationship between the expression of the ten hub genes and OS.

### Correlation of INK4 with immune cell infiltration and immune checkpoints

TIMER (https://cistrome.shinyapps.io/TIMER/) [18] was used to detect the correlation between the expression of INK4 and immune cell infiltration (macrophages, neutrophils, dendritic cells, CD4+ T cells, CD8+ T cells, and B cells). Moreover, the correlation of INK4 with immune checkpoints, including PD1, PD-L1, and CTLA-4, was evaluated in HCC using R with the ggplot2 package based on TCGA database. Statistical significance was set at *P*<0.05.

### Statistical methods

The R software (V.3. 6.3, The R Project for Statistical Computing, Vienna, Austria) was used for statistical analysis. The expression between different groups was compared using the Wilcoxon rank-sum test or paired sample *t*-test, as appropriate. Correlation analysis was performed using Pearson or Spearman correlation tests, as appropriate. Kaplan–Meier plots were drawn, and the log-rank test was performed to identify the significance of the difference between survival curves. Statistical analysis was automatically performed using the online database mentioned above. Statistical significance was set at *P*<0.05.

## Results

### High expression of INK4 genes in HCC

**Figure 1:**
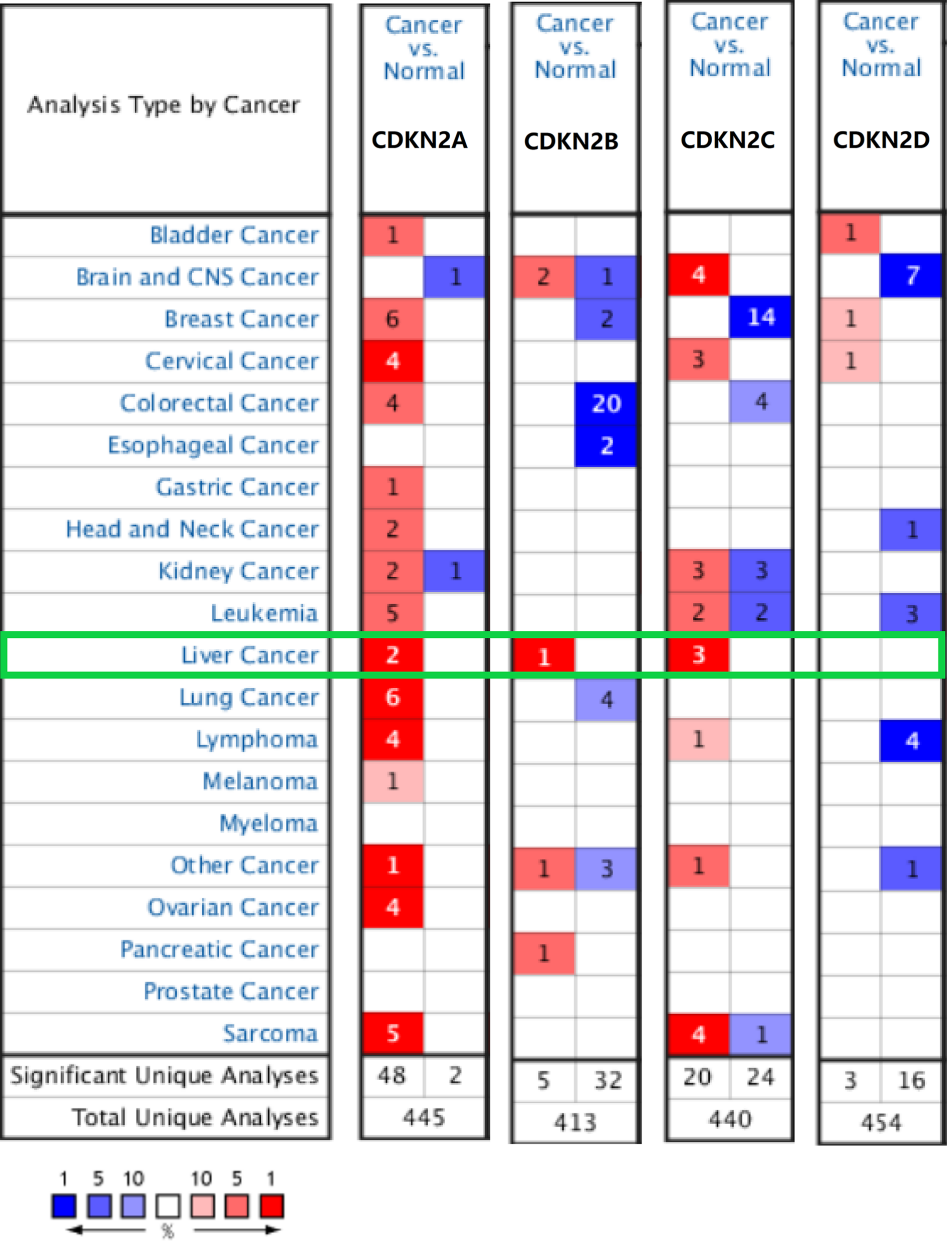
Transcription levels of INK4 family members in 20 different types of cancers in the ONCOMINE database Panels show the numbers of datasets with either significant up-regulation (red) or down-regulation (blue) of mRNA expression of the target genes. The threshold was designed using the following parameters: *P*-value of 0.01 and fold-change of 1.5.

**Figure 2:**
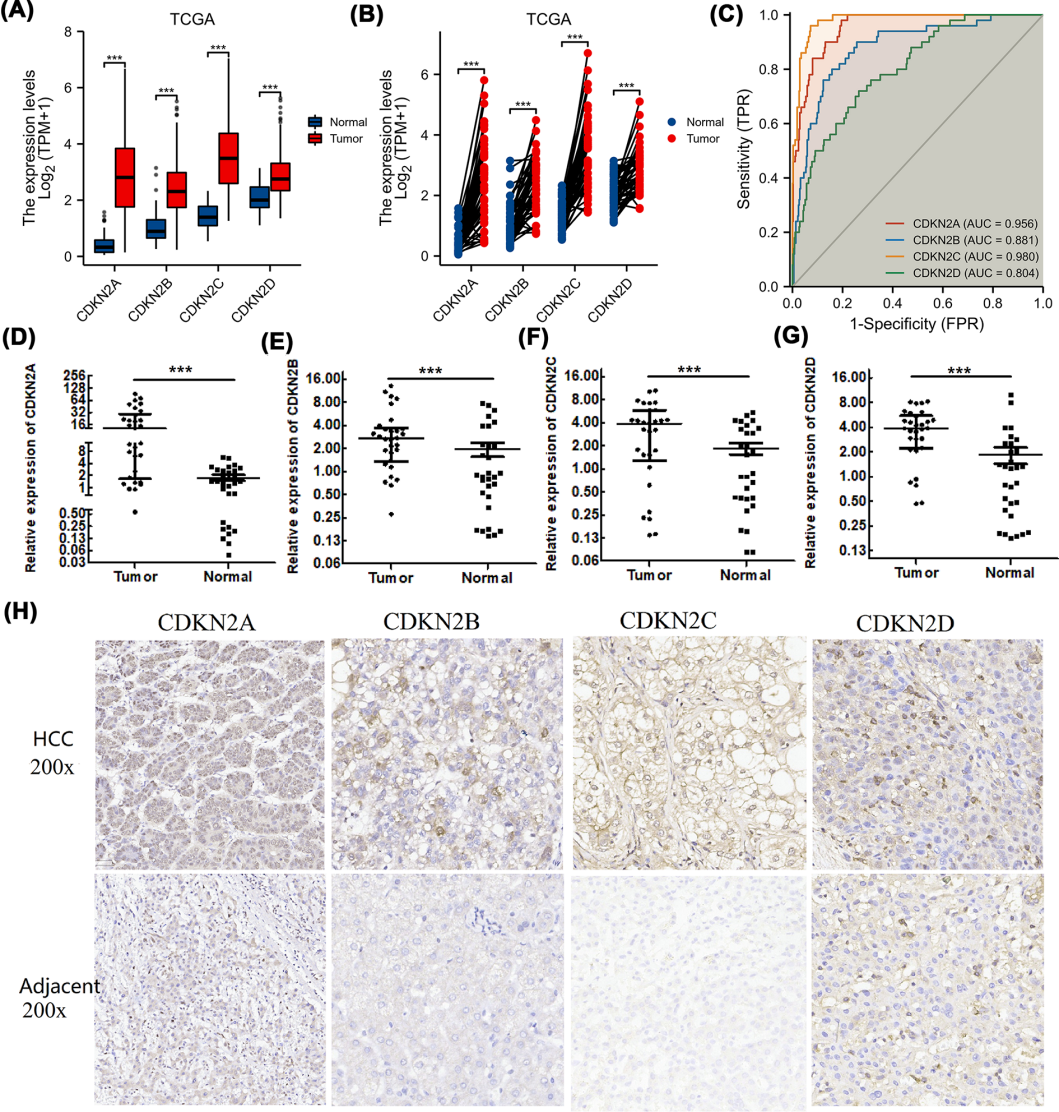
Overexpression of INK4 family in HCC (**A**) The expression levels of INK4 family in 374 HCC tissues and 50 normal liver tissues based on TCGA. (**B**) The expression levels of INK4 family in 50 HCC tissues and their paired adjacent normal liver tissues based on TCGA. (**C**) The ROC curve of INK4 family based on TCGA. (**D–G**) Quantitative PCR analysis of INK4 family members in clinical HCC samples. (**H**) Immunohistochemical-staining results of INK4 family members in clinical HCC samples. ***P<0.001.

The expression of CDKN2A, CDKN2B, CDKN2C, and CDKN2D was analyzed in 20 types of cancers. CDKN2A, CDKN2B, and CDKN2C were up-regulated in HCC (Figure 1). CDKN2A was overexpressed in HCC versus normal tissue in two datasets, with a fold-change of 4.276 and *P*-value of 6.59 × 10^−12^ for Wurmbach et al.’s dataset [19], and a fold-change of 2.347 and *P*-value of 2.17 × 10^−^^40^ for Roessler et al.’s dataset (Table 1) [20]. The expression of CDKN2B was significantly higher in HCC than in normal tissues, with a fold-change of 4.202 and *P*-value of 3.00 × 10^−11^ for Wurmbach et al.’s dataset (Table 1) [19]. CDKN2C expression was significantly higher in HCC in three datasets, with a fold-change of 3.335 and *P*-value of 1.02 × 10^−25^ in Chen et al.’s dataset [21] and fold-changes of 3.164 (*P*=4.55 × 10^−61^), and 2.216 (*P*=2.80 × 10^−5^) in Roessler et al.’s [20] and Roessler et al.’s [22] datasets (Table 1). We further compared the expression of INK4 between HCC and normal tissues based on data retrieved from TCGA database. CDKN2A, CDKN2B, CDKN2C, and CDKN2D were highly expressed in HCC in both paired and unmatched comparative studies (Figure 2A,B).

**Table 1:** Expression of INK4 family members in HCC from the Oncomine database

Name	Types of HCC vs. normal	Fold-change	*t*-test	*P*-value	Reference
CDKN2A	HCC vs. Normal	4.276	9.523	6.59 × 10^−12^	[19]
	HCC vs. Normal	2.347	15.934	2.17 × 10^−40^	[20]
CDKN2B	HCC vs. Normal	4.202	8.952	3 × 10^−11^	[19]
CDKN2C	HCC vs. Normal	3.335	12.263	1.02 × 10^−25^	[21]
	HCC vs. Normal	3.164	20.767	4.55 × 10^−61^	[20]
	HCC vs. Normal	2.216	4.892	2.80 × 10^−5^	[22]

HCC, hepatocellular carcinoma.

ROC curve analysis confirmed that CDKN2A, CDKN2B, CDKN2C, and CDKN2D had high diagnostic potential for HCC. The diagnostic potential of CDKN2A and CDKN2C was the greatest, as the areas under the curve were 0.956 and 0.980, respectively (Figure 2C). qRT-PCR (Figure 2D–G) and immunohistochemical staining (Figure 2H) of the clinical samples verified the higher CDKN2A, CDKN2B, CDKN2C, and CDKN2D expression in HCC than in adjacent normal liver tissues at both the mRNA and protein levels.

### Correlation of INK4 expression with cancer stage and prognosis

**Figure 3:**
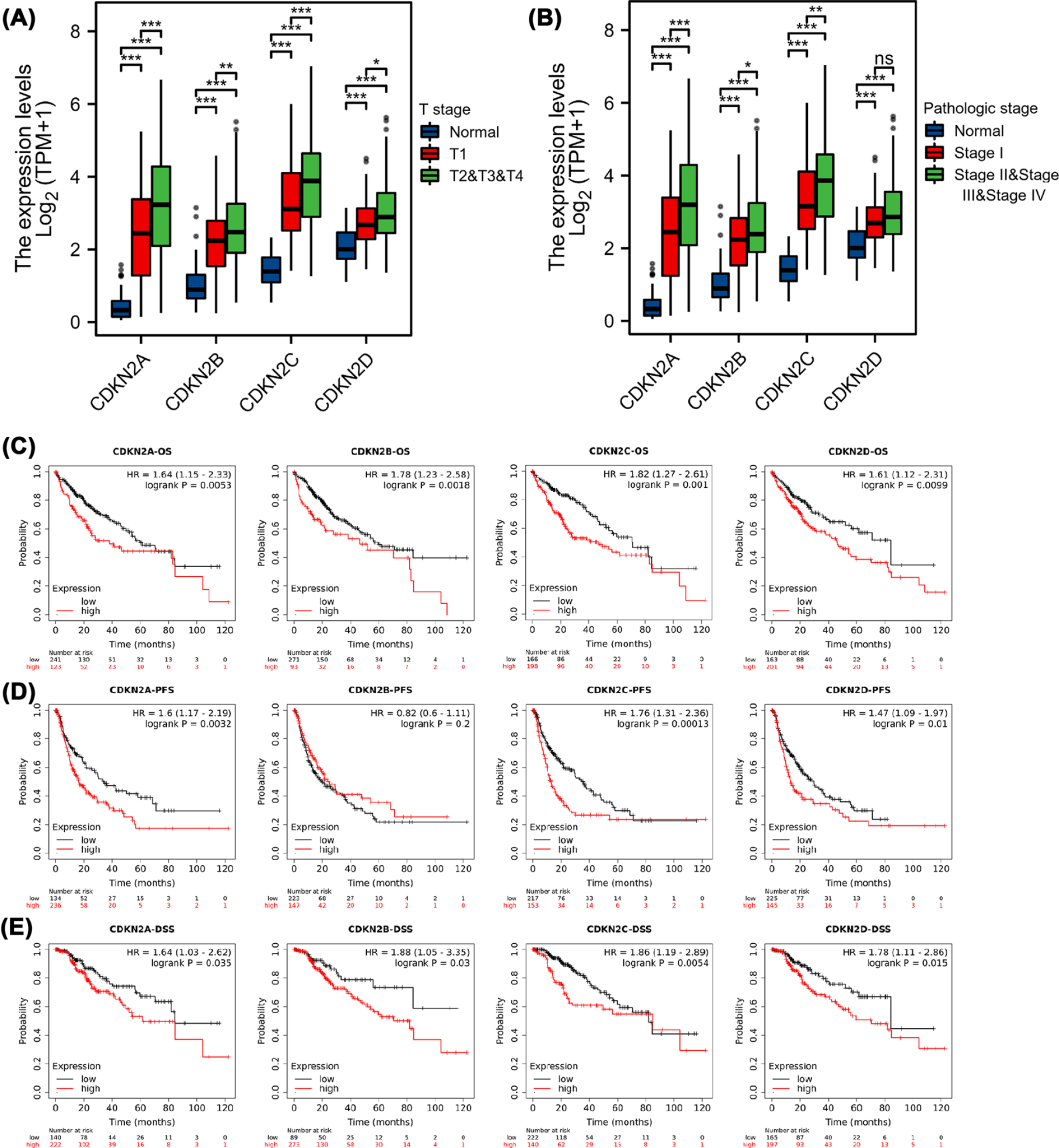
Relationship between mRNA expression of INK4 family and cancer stages and prognosis (**A**) The correlation analysis of mRNA expression of all INK4 family members and T stages of HCC. (**B**) The correlation analysis of mRNA expression of all INK4 family members and pathologic stage. The Kaplan–Meier survival curves for OS (**C**), PFS (**D**), and DSS (**E**). Comparing patients with high (red) and low (black) expression of INK4 family members in HCC.

We further analyzed the relationship between the expression of INK4 and cancer stage (T stage and pathological stage) and prognosis (OS, PFS, and DSS) of patients with HCC. The expression of CDKN2A, CDKN2B, CDKN2C, and CDKN2D was positively correlated with the T stage (Figure 3A), and the expression of CDKN2A, CDKN2B, and CDKN2C was positively correlated with the pathological stage (Figure 3B), indicating that patients with more advanced cancer tended to express higher mRNA levels of INK4. According to the Kaplan–Meier survival curves, a higher expression of CDKN2A, CDKN2C, and CDKN2D was corelated with worse OS, PFS, and DSS (*P*<0.05), and the expression of CDKN2B indicated poor OS and DSS (Figure 3C–E).

### Enrichment analysis of INK4-associated genes in HCC

**Figure 4:**
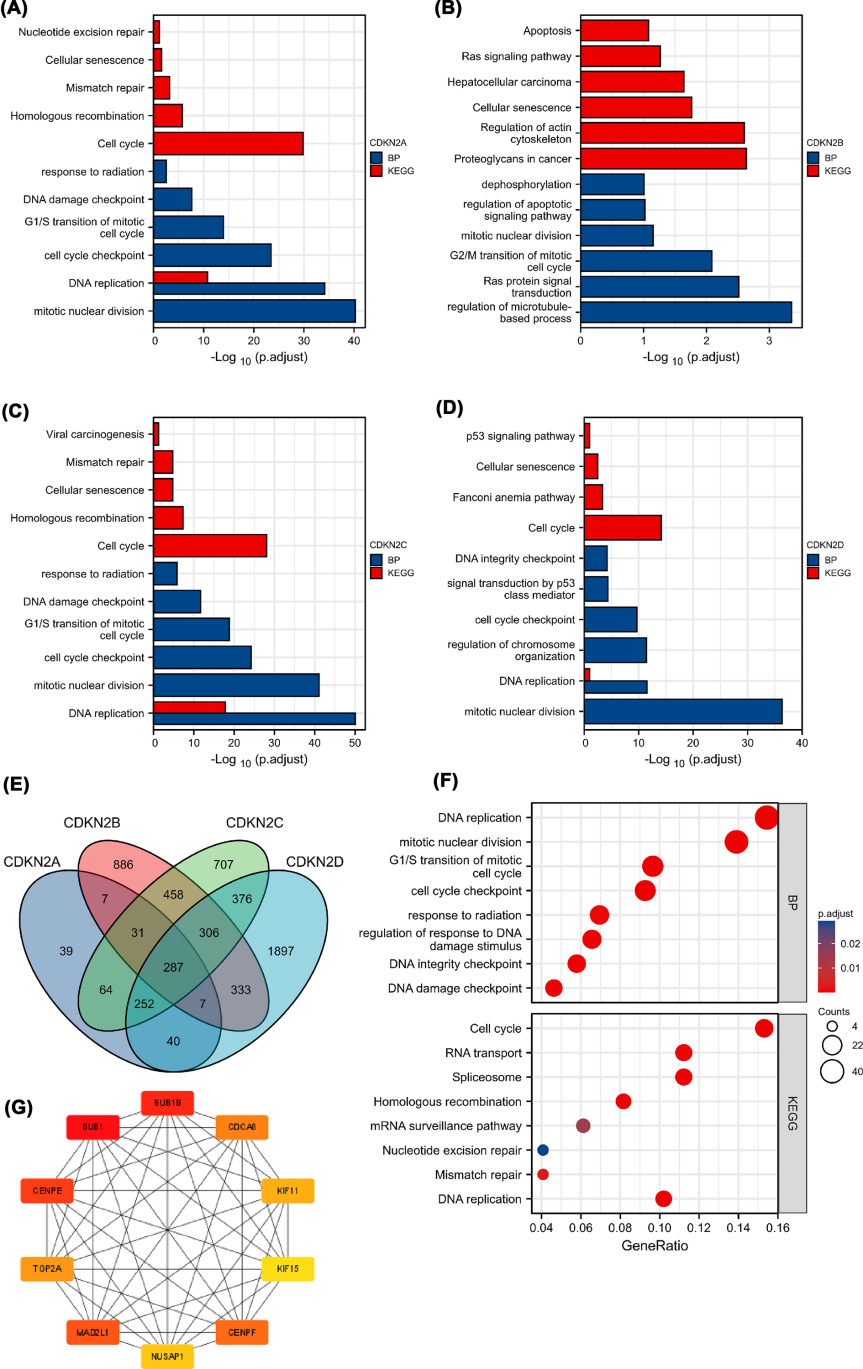
Biological process and KEGG pathway enrichment analysis (**A–D**) Biological process and KEGG analysis of all INK4 family members, including CDKN2A (**A**), CDKN2B (**B**), CDKN2C (**C**), and CDKN2D (**D**). (**E**) Venn diagram showing the intersection of coexpressed genes of INK4 family members. (**F**) Biological process and KEGG pathway enrichment analysis of intersected coexpressed genes of INK4 family members. (**G**) Ten hub genes selected using CytoHubba.

GO and KEGG analyses were performed for the top 200 correlated genes of INK4 to clarify their potential roles in HCC. GO and KEGG analyses suggested that CDKN2A, CDKN2B, CDKN2C, and CDKN2D were mainly involved in biological processes associated with cell-cycle regulation (such as cell-cycle checkpoint and G1/S transition), mitosis (including chromosome separation, mitotic mitosis, and spindle assembly), DNA replication, and DNA repair after damage (such as DNA integrity checkpoint, DNA damage checkpoint, and response to radiation) (Figure 4A–D). A total of 287 intersecting correlated genes (|r| > 0.4 and *P*<0.001) identified from the Venn diagram for subsequent GO and KEGG analyses were selected to predict common functions of the INK4 family (Figure 4E). The genes were mainly involved in cell-cycle regulation, mitosis, DNA replication, and DNA repair (Figure 4F). Ten hub genes were screened using CytoHubba: *BUB1, BUB1B, CDCA8, CENPE, CENPF, KIF11, KIF15, MAD2L1, NUSAP1, and TOP2A* (Figure 4G). These genes were mainly involved in cell-cycle regulation, mitosis, and DNA replication in gene annotation (Table 2). The Kaplan–Meier survival curve showed that all these hub genes were related to a lower OS in patients with HCC (Table 2).

**Table 2:** Annotation of ten intersecting coexpressed genes of the INK4 family and their prognostic value

Hub genes	Annotation	HR	*P*
*BUB1*	Mitotic checkpoint	1.75 (1.24–2.49)	0.002
*BUB1B*	Mitotic checkpoint	1.56 (1.10–2.21)	0.013
*CDCA8*	Key regulator of mitosis.	1.98 (1.39–2.82)	<0.001
*CENPE*	Microtubule plus-end-directed kinetochore motor	1.60 (1.13–2.26)	0.008
*CENPF*	Required for chromosome segregation	1.61 (1.14–2.28)	0.007
*KIF11*	Required for establishing a bipolar spindle	1.77 (1.25–2.52)	0.001
*KIF15*	Involved in mitotic spindle assembly	1.70 (1.20–2.42)	0.003
*MAD2L1*	Component of the spindle-assembly checkpoint	1.53 (1.08–2.16)	0.016
*NUSAP1*	Nucleolar and spindle-associated protein 1	1.52 (1.07–2.15)	0.018
*TOP2A*	DNA topoisomerase 2-α	1.75 (1.23–2.48)	0.002

HR, hazard ratio.

### Relationship between INK4 and immune cell infiltration

**Figure 5:**
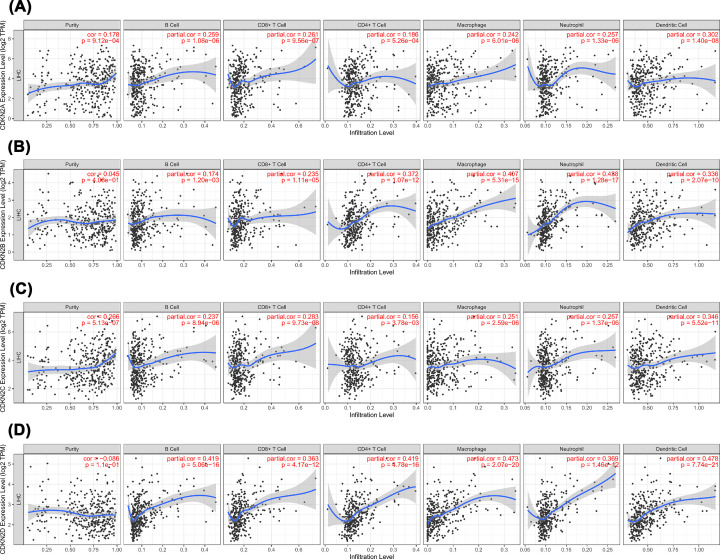
Associations between INK4 family members and immune cell infiltration in HCC (**A**) The correlation analysis of CDKN2A and the infiltration levels of six types of immune cells (macrophages, neutrophils, dendritic cells, CD4+ T cells, CD8+ T cells, and B cells). (**B**) The correlation analysis of CDKN2B and the infiltration levels of immune cells. (**C**) The correlation analysis of CDKN2C and the infiltration levels of immune cells. (**D**) The correlation analysis of CDKN2D and the infiltration levels of immune cells.

The relationship between INK4 and immune cell infiltration (macrophages, neutrophils, dendritic cells, CD4+ T cells, CD8+ T cells, and B cells) was investigated using TIMER. As shown in Figure 5, the expression of CDKN2A, CDKN2B, CDKN2C, and CDKN2D was positively correlated with the infiltration levels of six types of immune cells. CDKN2D showed the highest correlation with the infiltrating levels of macrophages (r = 0.473, *P*=2.07 × 10^−20^), neutrophils (r = 0.369, *P*=1.46 × 10^−12^), dendritic cells (r = 0.478, *P*=7.74 × 10^−21^), CD4+ T cells (r = 0.419, *P*=4.78 × 10^−16^), CD8+ T cells (r = 0.363, *P*=4.17 × 10^−12^), and B cells (r = 0.419, *P*=5.06 × 10^−16^).

### Relationship between INK4 and immune checkpoints

**Figure 6:**
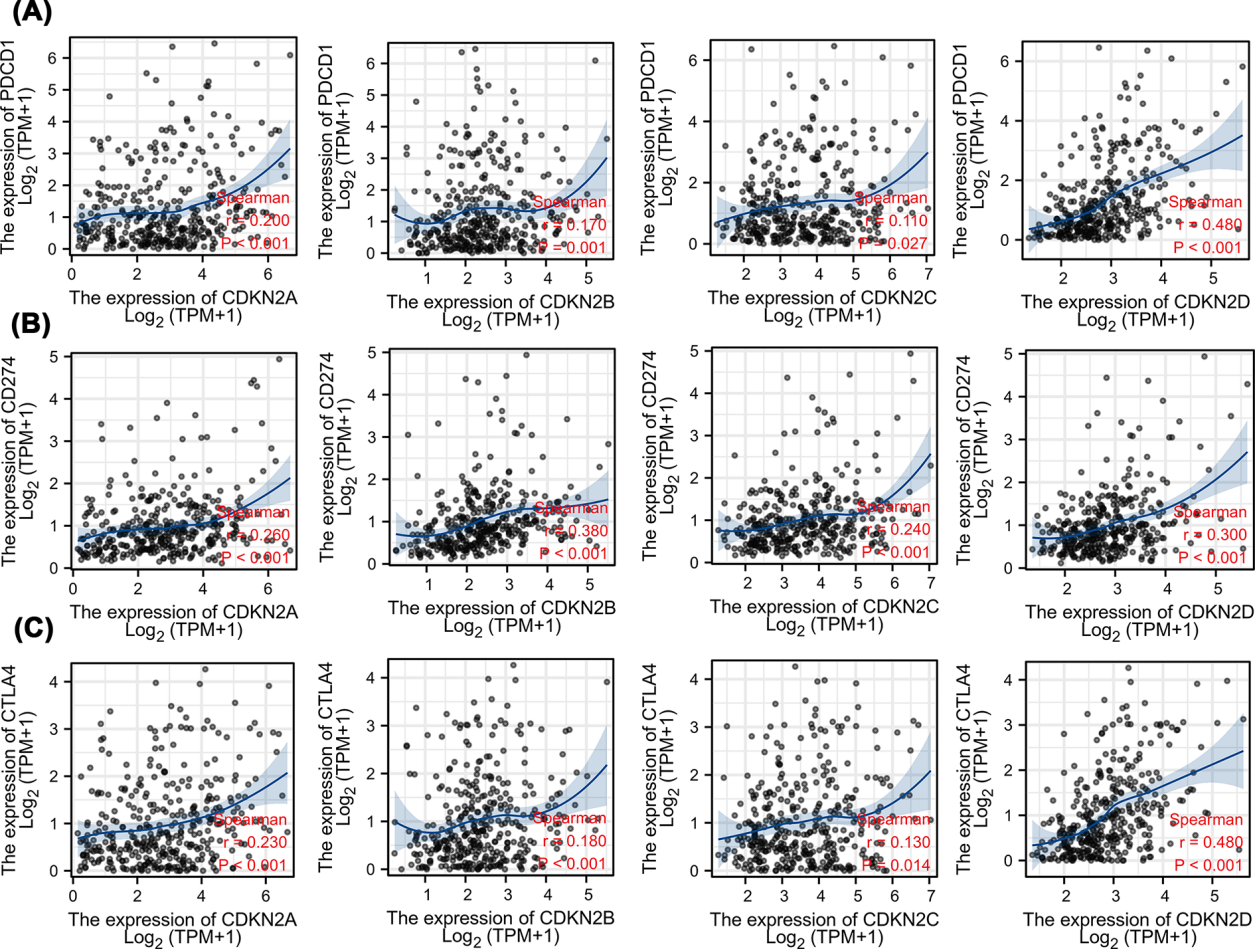
Correlation of the expression of INK4 family members with immune checkpoints in HCC (**A**) The correlation analysis of PD1 (PDCD1) and the expression of all INK4 family members. (**B**) The correlation analysis of PD-L1 (CD274) and the expression of INK4 family members. (**C**) The correlation analysis of CTLA-4 and the expression of INK4 family members.

CTLA-4, PD1 (PDCD1), and PD-L1 (CD274) are targets of immunotherapy, and immune checkpoint blocking of these sites has revolutionized the paradigm of cancer therapy. The relationship between INK4 members and PD1, PD-L1, and CTLA-4 was assessed in HCC (Figure 6). CDKN2A, CDKN2B, CDKN2C, and CDKN2D were positively correlated with PD1, PD-L1, and CTLA-4 (*P*<0.05), with CDKN2D showing the highest correlation with these immune checkpoints, PD-1 (r = 0. 480, *P*<0.001), PD-L1 (r = 0.3, *P*<0.001), and CTLA-4 (*r* = 0. 480, *P*<0.001).

## Discussion

CDKN2A is highly expressed in HCC and associated with prognosis and infiltrating levels of immune cells [12]; however, the role of the other INK4 family members, namely CDKN2B, CDKN2C, and CDKN2D, in the diagnosis, prognosis, and immune regulation of HCC is unclear. We comprehensively analyzed the roles of these other members in HCC. Based on analysis of both online public databases and clinical samples, the INK4 family members were overexpressed in HCC, which was closely related to an advanced cancer stage and poor prognosis. Along with CDKN2A, other INK4 family members, CDKN2B, CDKN2C, and CDKN2D, also showed a good ability to distinguish tumor from normal tissue, particularly CDKN2C, suggesting that they can be used as biomarkers for the screening, diagnosis, and prognosis prediction of HCC.

Previous researches have demonstrated the function of the INK4 family in cell-cycle regulation in HCC [23,24]. In addition to their roles as cell-cycle regulators, individual INK4 family members have been shown to perform diverse and distinct cellular tasks [8]. In the present study, GO and KEGG enrichment analyses revealed that the INK4 family members, together with their intersecting coexpressed genes, participated not only in cell-cycle regulation, including cell-cycle checkpoint and G1/S transition, but also in the biological processes of mitosis, DNA replication, and DNA damage repair, including DNA integrity checkpoint, DNA damage checkpoint, and response to radiation. INK4 proteins are commonly lost or inactivated by mutations in diverse types of cancer [25,26]. However, in our studies, hyperexpression of INK4 protein was increased in some patients with HCC [12,23,24]. A possible reason for this discrepancy is related to the functional role of INK4 proteins in the DNA damage repair pathway. Tumor cells exhibit genomic instability resulting from the accumulation of point mutations, deletions, and chromosome mis-segregations, which are not observed in normal cells, giving rise to deleterious DNA replication/repair stress and mitotic stress conditions that are lethal to cancer cells if left unchecked [27]. Cancer cells must tolerate this stress through support pathways [28]. Studies showed that after DNA damage, CDKN2A overexpression in tumoral cells caused cell-cycle arrest and inhibited genotoxic-induced apoptotic events. Blocking dysregulated cell-cycle progression can influence the sensitivity of the mitochondria to proapoptotic signals in DNA damage-induced cancer cells [29]. Furthermore, CDKN2D participates in DNA damage repair and plays a crucial role in regulating genomic stability and overall cell viability under conditions of genotoxic stress [8,10,30–34]. Basal levels of INK4 are transcribed in normal cells to monitor and quickly and efficiently repair sporadic alterations in chromatin that arise during the fine-tuned process of DNA replication. The sudden appearance of numerous aberrant DNA structures, which occurs in tumorigenesis, is accompanied by additional signals responsible for CDKN2D up-regulation [9,35,36], and abnormally high expression of CDKN2D inhibited DNA damage-induced apoptosis [34]. Marazita et al. [37] reported that the ATM-Chk2/ATR-Chk1 signaling pathways may be related to these functions.

HCC is an inflammation-related cancer, and its occurrence, progress, metastasis, and recurrence are closely related to the immune response [38,39]. Jiang et al. found that cell-cycle activity was associated with antitumor immunity in ten TCGA cancer cohorts, including HCC, and identified that the expression of many cell-cycle pathway genes was positively correlated with antitumor immunity [40]. The efficacy of immunotherapy requires sufficient immune infiltration into the tumor microenvironment and depends on the expression of immune checkpoint molecules [41,42]. Recent studies have reported that CDKN2A is closely associated with immune infiltration of HCC [12,43,44]. However, the relationship between other INK4 family members and HCC is unclear. We found that CDKN2A, CDKN2B, CDKN2C, and CDKN2D were not only positively correlated with the infiltration of immune cells, such as B cells, CD8+ T cells, CD4+ T cells, macrophages, neutrophils, and dendritic cells, but were also associated with immune checkpoints, including CTLA-4, PD1, and PD-L1, with high correlation coefficients between CDKN2D and the above-mentioned infiltrating immune cells and immune checkpoints. Previous studies showed that CDKN2D modulates the development of myeloid lineages, including megakaryocyte, erythroid, granulocytic, and macrophage lineages, and may also play a role in B-cell development [9,45]. Therefore, the role of the INK4 family, particularly CDKN2D, in the immune microenvironment of HCC should be further evaluated. These molecules may be useful as immunotherapy targets or improve the efficacy of other immunotherapy regimens.

In conclusion, we verified the diagnostic and prognostic predictive value of the INK4 family in HCC. The INK4 family not only participated in cell-cycle regulation but also inhibited genotoxic-induced apoptosis through the DNA damage repair pathway. Furthermore, the INK4 family was related to immune cell infiltration and immune checkpoints and may participate in the immune regulation of HCC. Therefore, the INK4 family is a potential diagnostic and prognostic biomarker and therapeutic target for HCC. It should be noted that our conclusion is mainly based on analyses of online databases (which is the main limitation of the present study), such as TCGA, and thus it should be verified by further molecular and animal experiments. Nevertheless, our study broadens the understanding of the INK4 family.

## Supplementary Material

Supplementary Tables S1-S2

## Data Availability

The datasets used and/or analyzed during the current study are available from the corresponding author on reasonable request.

## Abbreviations

CDKI: cyclin-dependent kinase inhibitor
DSS: disease specific survival
GAPDH: Glyceraldehyde-3-phosphate dehydrogenase
HCC: hepatocellular carcinoma
OS: overall survival
PFS: progression-free survival
qRT-PCR: Quantitative real-time polymerase chain reaction
ROC: receiver-operating characteristic
TCGA: The Cancer Genome Atlas
