# Supplementary material for: INK4 cyclin-dependent kinase inhibitors as potential prognostic biomarkers and therapeutic targets in hepatocellular carcinoma
Source: Biosci Rep. 2022 Jul 14;42(7):BSR20221082. doi: 10.1042/BSR20221082 (PMC9284345; doi:10.1042/BSR20221082)
Supplement: Supplementary Tables S1-S2 [file BSR-2022-1082_supp.pdf]

## Supplementary materials

**Table S1. Information of 30 patients with HCC selected from the Second Hospital of Dalian Medical University**

| Case | Gender | Age | Viral Hepatitis | TNM | Pathological stage | Tumor size (cm) | AFP (IU/mL) | Histologic grade | Cirrhosis |
|---|---|---|---|---|---|---|---|---|---|
| 1 | Female | 68 | HCV | pT1bN0M0 | IB | 3*2.3*1.7 | 4.9 | G2 | YES |
| 2 | Male | 51 | HBV | pT2N0M0 | II | 2.5*2*2.5 | 2.3 | G2 | YES |
| 3 | Male | 55 | HBV | pT2N0M0 | II | 2.2*1.5*2 | 14.9 | G2 | YES |
| 4 | Male | 53 | HBV | pT2N0M0 | II | 4*2.7*2.5 | 74.61 | G3 | No |
| 5 | Male | 50 | HBV | pT2N0M0 | II | 3.7*2.8*2 | 46.14 | G2 | YES |
| 6 | Male | 48 | HBV | pT3N0M0 | IIIA | 7.5*6*5.9 | 9.5 | G2 | YES |
| 7 | Male | 60 | HBV | cT2N0M0 | II | 4*3.2*3 | 5.9 | G3 | YES |
| 8 | Male | 63 | no | pT2N0M0 | II | 5.5*6.5*5 | 1260 | G3 | No |
| 9 | Female | 50 | no | pT2N0M1 | IVB | 10*8.8*5.5 | 44654 | G3 | No |
| 10 | Male | 52 | HBV | PT4N0M0 | IIIB | 13*11*6 | 96.7 | G3 | YES |
| 11 | Female | 57 | no | sT1bN0M0 | IB | 5.3*5*4 | 2.7 | G2 | YES |
| 12 | Male | 71 | HCV | pT1bN0M0 | IB | 5.3*4.5*5 | 3.96 | G2 | No |
| 13 | Male | 48 | HBV | pT3N0M0 | IIIA | 6*4*5.5 | 492.2 | G3 | YES |
| 14 | Male | 75 | no | pT1bN0M0 | IB | 3.5*2.8*2.5 | 28.1 | G2 | YES |
| 15 | Male | 66 | HBV | cT2N0M0 | II | 6*5.5*5 | 932 | G3 | YES |
| 16 | Male | 51 | HBV | PT2N0M0 | II | 2.7*2.6*1.7 | 76.17 | G2 | No |
| 17 | Female | 39 | HBV | PT1aN0M0 | Ia | 1.7*1.5*1.8 | 57.4 | G2 | YES |
| 18 | Male | 79 | no | PT3bNXM0 | IIIA | 8*6*6.5 | 7.7 | G2 | No |
| 19 | Male | 63 | HBV | pT1bN0M0 | Ib | 8.5*8*7 | 1.53 | G2 | No |
| 20 | Male | 49 | HBV | PT1aN0M0 | IA | 1.51*1.2*0.9 | 35.62 | G2 | YES |
| 21 | Male | 57 | no | PT1aN0M0 | IA | 1.6*1.4*1.5 | 17.2 | G2 | YES |
| 22 | Male | 64 | HBV | pT2N0M0 | II | 1.5*0.7*1.4 | 72.5 | G2 | YES |
| 23 | Male | 62 | HBV | pT4N0M0 | IIIB | 4.4*3.5*3.5 | 2758.66 | G2 | YES |
| 24 | Male | 80 | no | pT1bN0M0 | Ib | 4*3.7*3.5 | <1.08 | G1 | YES |
| 25 | Female | 69 | HBV | PT2N0M0 | II | 3*2*3.5 | 1.6 | G2 | YES |
| 26 | Male | 49 | HBV | PT2N0M0 | II | 8.5*6.5*12 | 10.86 | G2 | YES |
| 27 | Male | 77 | HBV | PT2N0M0 | II | 4.5*3.5*3 | 4.7 | G3 | YES |
| 28 | Male | 69 | HBV | PT2N0M0 | II | 5*4*5.5 | 5.7 | G2 | YES |
| 29 | Male | 76 | HBV | PT2N0M0 | II | 7.5*7*6 | 5.6 | G3 | No |
| 30 | Male | 71 | no | PT4N1M0 | IVa | 7.5*5.5*4 | 1609 | G2 | YES |

**Table S2. Primer sequences of INK4 members and GAPDH used for qRT-PCR**

| Gene | Primer | Sequences (5'-3') |
|---|---|---|
| | CDKN2a-F | GAAGGTCCCTCAGACATCCC |
| CDKN2A | CDKN2a-R | TGAAAACTACGAAAGCGGGGT |
| | CDKN2b-F | CGCGGGGACTAGTGGAGAA |
| CDKN2B | CDKN2b-R | GCGCCTCCCGAAACGGT |
| | CDKN2c-F | GGGGACCTAGAGCAACTTACT |
| CDKN2C | CDKN2c-R | CAGCGCAGTCCTTCCAAAT |
| | CDKN2d-F | TCACACTGCTGTGGTCAGCTTT |
| CDKN2D | CDKN2d-R | CGTCCCTGCGATGGAGAT |
| | GAPDH-F | CAGCCTCAAGATCATCAGCAAT |
| GAPDH | GAPDH-R | ATGAGTCCTTCCACGATACCAAA |
